# The challenging path to proving safety and effectiveness: A case of flow diverters in unruptured intracranial aneurysms

**DOI:** 10.1093/esj/23969873251369440

**Published:** 2026-01-01

**Authors:** Victor Volovici

**Affiliations:** Department of Neurosurgery, Erasmus MC University Medical Center, Rotterdam, The Netherlands; Center for Complex Microvascular Surgery, Erasmus MC University Medical Center, Rotterdam, The Netherlands

“Significant” *p*-values are overly-prevalent in scientific literature. The scientific literature dealing with medical devices hones in on a different incentive, that is, demonstrating that a device is “safe and effective.” The conclusions claiming that a device is “safe and effective,” sometimes in spite of the outcomes measured, are just as prevalent in intracranial aneurysm (IA) scientific literature, as significant *p*-values are in general medical literature.^1^

Demonstrating safety and effectiveness, however, is a much more difficult endeavor than simply reporting the results of a registry of treated patients or of a meta-analysis of registries.^1^ Flow diverting (FD) stents or pipeline embolization devices have constituted a major breakthrough in the treatment of unruptured IAs, with several studies in the early 2010s showing major promise of these new devices, particularly for side-wall or large internal carotid artery aneurysms. The U.S. Food and Drug Administration (FDA) approved FDs for “adults with large or giant wide-necked aneurysms in the internal carotid artery from the petrous to the superior hypophyseal segment.”

In 2022, the European Stroke Organisation (ESO) guidelines for the treatment of unruptured IAs were published.^2^ In this document, after review of the evidence, FDs were recommended against, for IAs where other treatment options existed. A backlash promptly appeared, with a letter to the editor making use of the leitmotiv “safe and effective.”^3^ The scientific underpinning of the “safe and effective” claim in that letter was a 2023 meta-analysis “of more than 1000 patients” published after the ESO guideline.^4^ A critical appraisal of this meta-analysis raises ample issues. Methodologically, the risk of bias of the studies was underestimated and neither statistical heterogeneity nor its causes were explored. Certain estimates lacked confidence intervals. Only radiological outcomes and long-term complications were reported. But perhaps most striking, the manuscript suggested biological plausibility through a “dose-response” in which the complete occlusion rates become progressively better on long-term follow-up. What is not stated is that while more than 1000 patients were included in the 1-year follow-up, fewer than a quarter were included in the 5-year follow-up.^4^ The cohorts pooled for the various follow-up timepoints are therefore not the same and the “dose-response” suggestion is somewhat misleading.

Within this scientific backdrop, it is highly commendable that the group in charge of the ESO guideline on unruptured IAs have performed a new meta-analysis on the current use and clinical outcomes of FDs.^5^ All studies included were uncontrolled. Perhaps the most interesting aspect, however, is the realization that of the 527 aneurysms for which size data were available, almost 90% were smaller than 10 mm. If we go back to the FDA approval, the scientific literature overwhelmingly deals with off-label indications of FDs, for which established treatment options already exist. One has to wonder, based on the complication rate reported in this meta-analysis, how FDs would fare in a head-to-head comparison with current available treatments and whether they would reach the non-inferiority threshold. The ESO guidelines recommend caution, and rightfully so.

Regardless of the results of the meta-analysis, FDs have been a major breakthrough in IA treatment. Which IAs primarily benefit from FD treatment is still a topic of debate, and hopefully this meta-analysis will not stir negative emotions, but a positive drive to perform prospective, comparative studies with a control group.

## Data Availability

Only published data was used and referenced
